# Compassion as Compass: Navigating 50 Years of Change and Challenge in Mental Health Nursing

**DOI:** 10.1111/jan.70120

**Published:** 2025-08-08

**Authors:** Carmel Bond

**Affiliations:** ^1^ School of Health and Social Care, Department of Nursing & Midwifery Sheffield Hallam University Sheffield UK

**Keywords:** compassion, history, mental health, nursing theory

## 1 Introduction

Reflecting on the evolution of mental health nursing, I think about the generations of nurses who paved the way, championing compassionate, recovery‐focused care. Our journey from custodial asylums to today's person‐centred practice is more than a historical shift; it is a testament to the enduring power of holism and relational connection in our field. Over more than 30 years working as a nurse, I have found that it is the quiet moments, those times when we simply listen without judgement or stand alongside someone in their most vulnerable hours that truly define our profession.

Over time, frameworks like CHIME and the Tidal Model have come to place individuals' stories and strengths at the heart of practice, inspiring hope, empowerment and meaningful partnerships. As someone who has dedicated my practice to patient‐centred care and my research to compassion, I see my own journey as both a continuation of this legacy and a commitment to shaping a future where every nurse is empowered to make a difference. Yet, pressures like staffing shortages and heavy workloads continue to test the profession's ability to stay true to its relational roots.

In this commentary, I trace key historical milestones and the philosophical, theoretical and contemporary challenges that have shaped mental health nursing. I will explore how compassionate connections have empowered service users, redefined professional identity and consider what is needed to sustain these values in the face of future challenges. Ultimately, this commentary asks what is needed to sustain compassionate, effective mental health nursing in an increasingly complex world.

## 2 Historical Context: Challenges and Debates

Fifty years ago, mental health nurses worked in asylums under strict medical hierarchies, where rigid routines often limited opportunities for meaningful patient connection (Nolan 2021). Prior to this, Hildegard Peplau, often referred to as ‘Mother of Psychiatric Nursing’ had introduced her Interpersonal Relations Theory (1952). Peplau's framework for ‘therapeutic’ nurse–patient relationships emphasised trust‐building phases like ‘Orientation’ (Forchuk 2024).

While Peplau's theory laid the groundwork for a more person‐centred, compassionate approach, putting these ideals into practice proved challenging (Jojan and Carroll 2024); the realities of institutional care often made such therapeutic engagement difficult. However, these very challenges prompted a critical re‐examination of nursing's role, leading to the development of new knowledge that placed the nurse–patient relationship at the centre of practice (Haber 2000).

Peplau's theory was revolutionary in shifting nursing from a custodial, task‐oriented approach to one focused on interpersonal connection, communication and mutual growth, laying a foundation for future advances in person‐centred care. Through his extensive writings, outlining his lived experiences of working in the asylums, Peter Nolan, Emeritus Professor of Mental Health Nursing, described the challenges in advancing person‐centred principles (Nolan 2021). His accounts provide valuable context for understanding the realities faced by patients and staff from the 1960s onward, and the difficulties in providing person‐centred care.

As Nolan (2021, 2022) illustrates, mid‐20th century psychiatric care often fell short of Peplau's aspirations due to institutional constraints that limited nurses’ ability to deliver individualised care. Nurses worked in chronically overcrowded, under‐resourced environments governed by rigid routines, with little differentiation between patients. Recent research has shown staffing shortages and high clinical workloads continue to challenge nurses’ ability to build therapeutic connections and work in person‐centred ways (Kwame and Petrucka 2021). Nonetheless, mental health nurses have continued to prioritise the approach set forth by Peplau—by striving to forge meaningful connections to enhance patient care (Painter and Bond 2023; Bond, Hui, et al. 2022).

Central to these connections is the language used to describe those receiving care, as terminology itself can shape the nature and quality of the nurse–person relationship. Over the years there has been a continuing debate about terms used to refer to people who use mental health services, particularly the use of the term ‘patient’ versus alternatives such as ‘service user’, ‘client’ or ‘person receiving care’. This debate is complex and deeply consequential, reflecting not only changing trends in person‐centred care but also deeper questions of power, agency, and professional boundaries (Jackson et al. 2016).

Critics of ‘patient’ argue that it reinforces passivity, medical dominance, and hierarchical relationships, potentially undermining personhood and limiting shared decision‐making (Jojan and Carroll 2024; Stacey et al. 2016). This has led to the adoption of terms like ‘service user’ or ‘survivor,’ which aim to foreground autonomy and lived experience, particularly among those who have felt marginalised by traditional services.

Conversely, proponents of ‘patient’ emphasise its role in clarifying the therapeutic relationship and safeguarding professional boundaries, thus protecting the well‐being of those seeking care. As Jackson et al. (2016) note, ‘patient’ is a temporary, context‐specific identity that helps ensure the primacy of care and reduces the risk of role confusion.

Ultimately, preferences for terminology are highly individual and context dependent. Some may value the familiarity and legitimacy of ‘patient,’ especially in settings where parity with physical health care is important. Others, particularly those with negative experiences of institutional care, may prefer terms emphasising autonomy and agency—while also resisting stigma (Lyon and Mortimer‐Jones 2020; Simmons et al. 2010).

Despite ongoing debate, there is a notable lack of contemporary, empirical research on terminology preferences in mental health care, from a first‐person perspective. This gap underscores the need for up‐to‐date, inclusive research to inform best practice. For now, a nuanced approach is warranted—one that respects individual and cultural preferences, maintains clarity of roles and boundaries, recognises the ethical and practical implications of terminology, and engages all stakeholders to ensure language reflects both personhood and the sanctity of care (Jackson et al. 2016). Ultimately, compassionate, person‐centred care requires flexibility and respect for individual preferences, rather than rigidly adhering to or imposing any single term as universally appropriate (Bond, Hui, et al. 2024).

The continuing debate around terminology reflects the profound influence of language in shaping care relationships, professional identity, and ethical practice. This complexity around terminology is not merely semantic; it is deeply entwined with the historical realities of care. Nolan (2021) describes how the conditions of the asylums often meant routine care took precedence over patients' individual needs. Nurses were frequently caught between the needs of patients and the decisions of doctors, their autonomy constrained by a strict medical hierarchy. These institutional realities, alongside the ongoing debates about the language of care and the evolving understanding of the nurse–patient relationship, set the stage for further transformation in mental health nursing. As mental health care entered the twentieth century, these dynamics contributed to the emergence of new paradigms, most notably the biomedical model.

Throughout much of the twentieth century, the biomedical model dominated mental health care, prioritising pharmacological interventions and often overlooking psychological, social, and environmental factors (Deacon 2013). However, as criticism of the biomedical model grew and political momentum shifted toward community‐based care, the UK's Community Care Act 1990 (National Health Services 1990) signalled a significant policy turning point. This legislation closed asylums and aimed to shift care into the community, based on the belief that nurses, formerly cast in custodial roles resembling ‘prison wardens’, could now foster stronger therapeutic relationships and exercise greater assessment and advocacy skills (Sidi 2022). While this policy provided new opportunities for professional autonomy and person‐centred practice (Nolan 1998), subsequent national evaluations revealed inconsistent implementation, shaped by local resources, and leadership priorities, which ultimately impacted the realisation of person‐centred care across different mental health trusts (Callaghan et al. 2012).

Some areas noted improvements in assessment and care planning, while others struggled with under‐resourcing, fragmented services, and the persistence of institutional practices in new forms, sometimes described as ‘mini asylums’ (Calabria and Cullen 2024). Critics have also noted that underinvestment and inconsistent implementation have, at times, led to inadequate support and a lack of access to meaningful therapeutic relationships for those with enduring mental health needs. As such, while deinstitutionalisation created opportunities for greater autonomy and advocacy within nursing, it also introduced new challenges and highlighted the need for sustained investment and reform in community‐based mental health services (Calabria and Cullen 2024).

Against this backdrop of mixed progress and persistent challenges, new models of care and philosophies began to emerge that sought to address the gaps left by deinstitutionalisation. One of the most influential of these was the recovery model, which gained prominence in the late 1980s and early 1990s (Davidson et al. 2021). Primarily driven by the consumer/survivor/ex‐patient movement in the United States, this grassroots initiative advocated for self‐help, patient rights and a greater focus on personal narratives and community support.

The recovery model emphasised hope and self‐determination, positioning relationships as central to mental health care (Slade and Longden 2015; Anthony 1993). This shift catalysed a redefinition of professional identity for mental health nurses. The recovery approach profoundly influenced my nursing philosophy, leading me to view my role not solely as a provider of care, but as a partner in patients' recovery journeys, supporting their autonomy and fostering hope through meaningful, therapeutic relationships.

This relational 'recovery oriented' shift enabled nursing to promote social inclusion, enhancing patients' lives (Kolarič et al. 2024). Consequently, nurses' relationships, working 'with' patients, have become crucial to recovery in mental health settings (Bond, Stickley, and Stacey 2024).

…by being invited in someone's life to share those things, you automatically open yourself to being a compassionate person (Bond, Hui, et al. 2022, 1190)

Yet, this deeply relational and compassionate aspect of mental health nursing stands in marked contrast to the profession's historical struggle for autonomy and identity. Throughout history, mental health nurses have faced ongoing challenges in defining their professional roles (Morrall 1998), largely due to the widely discussed influence of medical dominance and the notion that nurses serve in a subordinate or supportive capacity to doctors—often referred to as fulfilling a ‘handmaiden’ function (Buchanan‐Barker and Barker 2005).

However, narratives emphasising the power of compassion to enhance health outcomes are reshaping this traditional nurse–doctor relationship, especially within mental health nursing (Malenfant et al. 2022). Here, nurses see themselves as applying their own compassionate selves 'therapeutically' to support patient recovery—thereby positioning themselves as central providers of both care and treatment, rather than as subordinates within medical hierarchies (Bond, Hui, et al. 2022).

Building on this expanded relational identity, mental health nurses have developed increased independence, adaptability, and leadership in their practice. No longer confined to institutional walls, they now work across diverse settings, fulfilling complex roles as advocates, educators, and clinicians. They provide crisis intervention, promote health, and offer psychosocial support that spans individual, family and community needs. Painter (2021) refers to contemporary mental health nurses as ‘bio‐psycho‐social specialists,’ who act as prescribers while considering individuals' social, cultural and spiritual needs, remaining grounded in Peplau's therapeutic relational theory and person‐centred care.

These developments in nursing practice and identity have not only transformed mental health nursing but also contributed to a more collaborative and holistic approach across the wider healthcare system. Working closely with multidisciplinary teams, including psychiatrists, psychologists, social workers, and occupational therapists, mental health nurses now play a pivotal role in integrated care planning, advocacy, and the promotion of recovery‐oriented practice across services (Duyilemi and Mabunda 2025). This interprofessional collaboration has strengthened the quality and continuity of care, while also elevating the voice and expertise of nursing within the broader mental health landscape (Cleary et al. 2019).

As the profession continues to evolve, the challenge remains: how can mental health nurses sustain compassionate, person‐centred relationships in the face of ongoing systemic and societal change?

## 3 Philosophical and Theoretical Influences

As mental health nurses have redefined their roles through compassionate practice, broader philosophical and theoretical developments, such as the emergence of the critical psychiatry movement, have simultaneously influenced the field. The critical psychiatry movement, as a distinct and organised perspective, began to take shape in the late 20th century. The Critical Psychiatry Network (CPN) in the UK was formally established in 1999 by a group of British psychiatrists, but critical psychiatry's intellectual roots can be traced to earlier influential critiques, such as R.D Laing's anti‐psychiatry movement in the 1960's and the work of David Cooper and others, who challenged reductionism and positivism in mainstream psychiatry (Stacey and Bond 2025). In the U.S. Thomas Szasz was equally influential in his views, which contested the diagnostic classification of mental distress as an 'illness'.

Expanding on these critiques raised by the critical psychiatry movement, the concept of recovery has been transformed from a clinical outcome into a personal journey defined by hope, self‐determination, supportive relationships and social inclusion, a perspective long advocated by service user movements (Slade and Longden 2015). Contemporary research on compassion has continued to advocate for similar changes, emphasising the centrality of compassionate care as a foundation for recovery and as a means of fostering genuine partnership with patients (Bond et al. 2025).

This evolving approach to recovery is exemplified by the CHIME framework (Connectedness, Hope, Identity, Meaning, and Empowerment) (Health Services Executive 2018) and the Tidal Model (Buchanan‐Barker and Barker 2005; Stacey and Bond 2025). Both frameworks further illustrate this evolution by placing individuals' stories and strengths at the core of practice. Internationally, the Tidal Model has been shown to foster hope, collaboration, and improved outcomes for both service users and staff (Cook et al. 2005). These frameworks have profoundly shaped how I have approached mental health nursing. In my practice, working compassionately involves creating environments where individuals feel heard, valued and empowered to define their own path to recovery. My personal commitment to these models has strengthened my belief in the importance of hope, dignity and meaningful relationships as essential elements of effective mental health care.

However, the advancement toward recovery‐oriented and relational practice has not been without tension. The increasing focus on risk assessment and management, often referred to as the ‘risk agenda’, can sometimes conflict with the principles of recovery and person‐centred care, especially when risk management practices reinforce power imbalances or restrict patient autonomy (Ahmed et al. 2021). Mental health nurses must continually navigate these tensions, striving to balance safety with empowerment in therapeutic relationships.

At the same time, changing social attitudes, shaped by mental health promotion campaigns and anti‐discrimination legislation such as the Equality Act 2010, have challenged stigma and promoted inclusion (Henderson et al. 2020). These societal shifts have reinforced the importance of rights‐based, person‐centred approaches, supporting nurses as advocates for social justice and patient empowerment.

Whilst the historical significance of biomedical interventions and medications in mental health care is undeniable, it is the evolution of relational approaches, grounded in therapeutic engagement, empathy and collaboration, that has most profoundly redefined mental health nursing. Extensive research demonstrates that compassionate, person‐centred relationships are not simply complementary to treatment but are often the decisive factor in promoting recovery, fostering hope, and supporting long‐term well‐being (Bond, Hui, et al. 2024). The quality of the therapeutic relationship is consistently identified as central to positive outcomes, with compassionate practice providing a foundation for trust, empowerment and genuine partnership (Bond, Hui, et al. 2022).

These transformative developments have shifted the profession away from custodial or purely clinical models, positioning nurses as key advocates and collaborators within multidisciplinary teams. Despite practising in an increasingly complex world, therapeutic relationships and compassionate practice continue to overlap, creating the conditions for recovery and meaningful change (Wildbore et al. 2024). But a pressing challenge remains: how can these relational values be sustained and prioritised amid intensifying pressures from risk management agendas, resource constraints and the persistent influence of biomedical paradigms? (NHS England 2022; World Health Organization 2022).

## 4 Future Challenges and Opportunities

As the profession looks to the future, sustaining compassionate, relational care will require a renewed focus on the well‐being of mental health nurses themselves. Embedding well‐being into the fabric of mental health nursing, through education, leadership and policy, will be vital to ensuring that compassionate care remains sustainable, authentic, and transformative for all.

Sustaining relational practice requires that nurses feel valued, supported, and emotionally resilient. Prioritising initiatives such as reflective supervision, peer support and accessible mental health resources can help nurture the well‐being of nursing staff. Addressing systemic challenges, like staffing levels, workload and workplace culture, further strengthens the foundations for compassionate 'therapeutic' relationships with patients.

Reflective practice is widely recognised as foundational to professional development and staff well‐being in mental health nursing across international contexts (International Council of Nurses 2021). Engaging in regular, structured reflection, individually or as part of clinical supervision, enables nurses to process the emotional demands of their work, develop self‐awareness and strengthen their relational skills in complex situations (Stacey et al. 2020).

Clinical supervision is valued as a psychologically safe space that supports both skill development and emotional well‐being for mental health nurses. Recent reviews indicate that clinical supervision is generally associated with positive workforce outcomes, including enhanced competence, improved workplace culture, and greater compassion (McDonough et al. 2025). It is also important to note that the effectiveness of supervision may depend on factors such as the type of supervision, organisational culture, and staff attitudes regarding the perceived value of supervision (Martin et al. 2021).

Organisations must advocate for embedding reflective practice into healthcare systems globally, ensuring that all staff have access to psychologically safe environments for reflection, learning, and growth. This is critical because, when nurses have space to care for their own well‐being, they are better equipped to form genuine, healing connections with those they support (Jarden et al. 2021). For instance, structured reflective practice, as exemplified by the Nightingale Frontline leadership support service, has been shown to support staff well‐being and compassionate leadership internationally (Bond, Stacey, et al. 2022).

Investing in nurse well‐being is not only an ethical imperative but also essential for fostering the relational connections that empower recovery and inclusion for service users. To sustain compassionate, relational practice, organisations should prioritise structured reflective supervision, foster peer support initiatives and maintain adequate staffing to safeguard nurses' emotional health. Integrating these supports into policy and education will help secure the profession's relational foundations for the future. In this way, the future of mental health nursing rests on a commitment to compassion that begins within the profession and radiates outward, shaping recovery‐focused care for all.

## 5 Conclusion

Across historical, community, and theoretical perspectives, the centrality of compassion and therapeutic relationships has emerged as defining elements in the evolution of mental health nursing—from its origins in institutional control to today's relationship‐driven, person‐centred practice. This enduring focus continues to shape professional identity and the future direction of care. As I reflect on the generations of nurses who have championed empathy and advocacy, I am reminded that their legacy inspires both my own practice and that of colleagues worldwide.

For this evolution to continue, it is essential that nurses are supported through opportunities for reflective practice, access to resources and organisational cultures that prioritise well‐being. Sustaining compassionate, relational care in the face of ongoing systemic pressures, such as staffing shortages, risk management and the influence of biomedical paradigms, will require collective advocacy and leadership at every level. Placing compassion at the centre of practice and challenging barriers to therapeutic connection, mental health nurses can help forge an inclusive, empowering future for the profession and those they serve.

## Conflicts of Interest

The author declares no conflicts of interest.

## Data Availability

Data sharing is not applicable to this article, as no new data were created or analyzed in this study.
